# CO_2_ production, dissolution and pressure dynamics during silage production: multi-sensor-based insight into parameter interactions

**DOI:** 10.1038/s41598-017-14187-1

**Published:** 2017-11-07

**Authors:** Menghua Li, Guilin Shan, Haiyang Zhou, Wolfgang Buescher, Christian Maack, Kerstin H. Jungbluth, André Lipski, David A. Grantz, Youheng Fan, Daokun Ma, Zhongyi Wang, Qiang Cheng, Yurui Sun

**Affiliations:** 1 College of Information and Electrical Engineering, China Agricultural University, Key Lab of Agricultural Information Acquisition Technology, Ministry of Agriculture, 100083 Beijing, China; 2 Department of Agricultural Engineering, The University of Bonn, 53115 Bonn, Germany; 3 Institute of Nutrition and Food Science, The University of Bonn, 53115 Bonn, Germany; 4 Department of Botany & Plant Sciences, University of California at Riverside, Kearney Agricultural Center, Parlier, CA 93648 United States

## Abstract

Silage is a critical global feedstock, but is prone to aerobic deterioration. The dominant mechanism of O_2_ transport into silage remains unresolved. Here, multiple sensors tracked O_2_ and CO_2_, gas pressure (*ΔP*) between internal silage and ambient air, pH and silage temperature (T_si_) during the ensilage of maize and ryegrass. We report the first observation that CO_2_ produced from microbial respiration was partially dissolved in silage water, with evidence of negative or positive *ΔP* depending on the changing balance between CO_2_ production and dissolution. The *ΔP* < 0 reflected an apparent respiratory quotient (RQ) < 1. Net CO_2_ production was much greater in anaerobic fermentation stage than in initial aerobic phase or later aerobic feed-out phase. O_2_ transport into silage is intimately linked to the dynamics of net CO_2_, *ΔP*, microbial activity, pH and T_si_. These results suggested that both gas diffusion (based on Fick’s law) and advective transfer (Darcy’s law) play equally important roles in governing the complex temporal progression of inward and outward gas fluxes to and from the silage interior. Even though low pH suppressed microbial activity and supported aerobic stability, the negative *ΔP* increased the risk of O_2_ entry and aerobic deterioration during feed-out phase.

## Introduction

Silage is an important preservation technique, used world-wide to provide a nutritious and palatable animal feed months after harvest^1^. It is estimated that 200 million tons of dry matter (DM) are ensiled worldwide annually^1,2^. A significant fraction of the nutritive value is lost due to biological oxidation.

The biochemical production of silage relies on lactic acid anaerobic fermentation. The silage-making process is generally divided into four temporal phases: an initial aerobic phase, a fermentation phase, a prolonged storage phase, and a final feed-out phase^3^. The fermentation and storage phases are dominated by anaerobic processes. As a result, silage is susceptible to oxidation because it contains aerobic bacteria, yeasts and molds (fungi) that are dormant under anaerobic conditions. Introduction of oxygen (O_2_) enables these aerobic microorganisms to be quickly activated, producing carbon dioxide (CO_2_) and water through aerobic respiration^3–5^ and inducing loss of dry matter and nutrients, i.e. “spoilage” of the silage^3,6,7^. During feed-out, when the silo is opened for feeding to livestock, the face of the silage is unavoidably exposed to the oxidizing atmosphere and aerobic losses can be substantial^3^.

The amount of O_2_ present within the silage is affected by numerous competing factors, including silage density or porosity, moisture content, wind velocity and direction, thermal buoyancy and gradients in gas concentration, density and pressure^4,8,9^. Two physical mechanisms of O_2_ transport to the silage interior during ensilage and storage have been identified as gas diffusion, which is governed by the concentration gradient between the silage pore space and ambient air, and advective transfer, driven by differential gas pressure (*ΔP* = *P*_*air*_ − *P*_*silage*_) across the bunker silo seals. The first may be modelled based on Fick’s law^4,8,10–14^, and the second based on Darcy’s law^15–18^. Despite this mathematical tractability, the dominant mechanism for transport of O_2_ into the silage remains in question^7^.

While the biochemical process of silage-making has been mostly characterized, determination of conditions within the developing silage has been challenging. For gas composition and concentration inside the silage, a common method has been to extract gas samples from the silage for later analysis using a laboratory gas chromatograph^8,18,19^. Despite sufficient accuracy of the gas chromatography method, two technical issues are encountered. First, sample extraction may result in additional O_2_ entering the silage. Second, the method is time consuming and thus unable to track the dynamics of silage gasses. Measurement of pH within the silage during its solid-state fermentation has also been difficult to achieve^20^.

Despite these challenges, obtaining and characterizing these process level data will contribute to understanding and improving silage yield and quality. Here we present the first multi-sensor-based, simultaneous measurements of internal O_2_, CO_2_, *ΔP*, pH, and internal silage temperature (T_si_) over the whole silage-making process. The major objective of this study was to reveal the previously unknown dynamic interactions of the key parameters governing silage development and quality.

## Results

### Fresh plant materials (Test-A)

**Figure 1:**
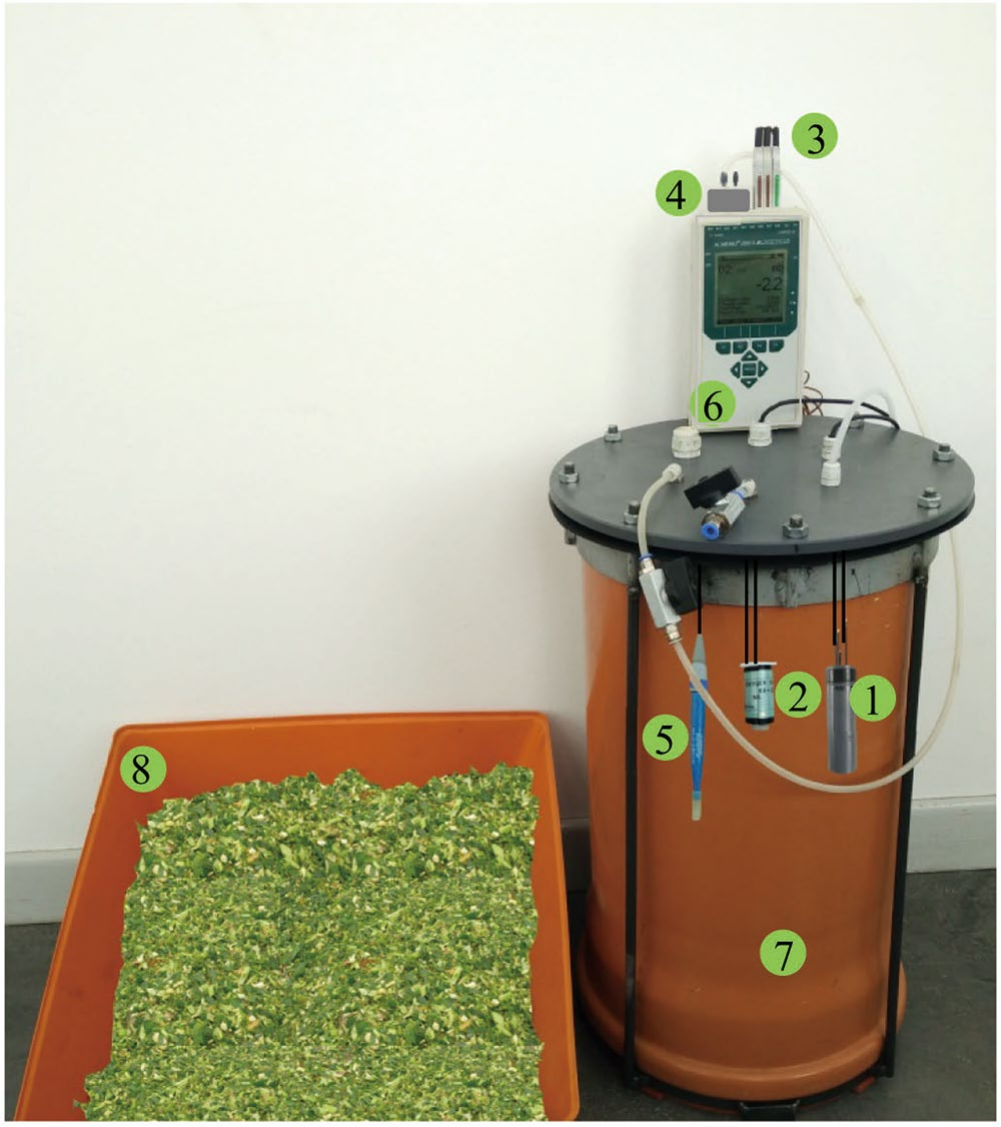
The experimental test silo for both Test-A and Test-B, which included ① CO_2_ sensor ② O_2_ sensor ③ temperature sensor ④ pressure sensor, ⑤ pH sensor, ⑥ ALMEMO data-logger ⑦ air-tight cylinder and ⑧ test substrate (here fresh chopped maize).

A unique suite of sensors was placed in a test silo (Fig. 1) such that naturally occurring changes could be monitored under realistic gas-tight conditions. Details are presented in Methods (below). The testing period consisted of the initial aerobic phase (about 3 h) and the anaerobic fermentation period (about 100 h), repeated three times with each of two fresh materials (maize and ryegrass).

**Figure 2:**
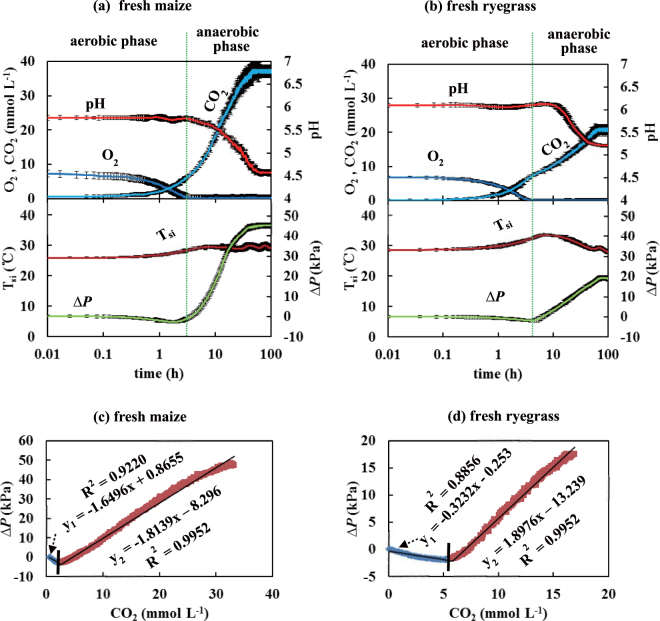
The time courses (means ± s.e.) of O_2_ and CO_2_ concentrations, pH, *ΔP* (inside to ambient) and internal silage temperature, T_si,_ during Test-A, the ensilage of fresh-chopped maize (Fig. 2a) and fresh-cut ryegrass (Fig. 2b). Note the logarithmic time axis. The relationships between *ΔP* and CO_2_ shown as piecewise linear regressions in Test-A during ensilage of fresh maize (Fig. 2c) (R^2^ = 0.9220, *p* = 5.94 × 10^−40^; R^2^ = 0.9952, *p* = 7.12 × 10^−80^), and fresh ryegrass (Fig. 2d) (R^2^ = 0.8856, *p* = 3.57 × 10^−61^; R^2^ = 0.9952, *p* = 3.70 × 10^−90^) in Test-A.

The time courses of O_2_, CO_2_, pH, *ΔP* and T_si_ (three independent replicates; Test-A) and relationships between *ΔP* and CO_2_ in Test-A were obtained from fresh-chopped maize (Fig. 2a,c) and fresh ryegrass (Fig. 2b,d). During the aerobic period, the initial pH was 5.8 for maize and 6.1 for ryegrass (Table 1; Fig. 2a,b), which was conducive to high activity of microorganisms and plant proteolytic enzymes^5,8,18,21^. While O_2_ was rapidly depleted to near zero in each test silo, the increase in CO_2_ was minimal.

**Table 1:** Physical properties, pH and counts of microorganisms of the tested materials. Values marked ND are below the level of detection.

		Fresh BD (kg m^−3^)	DMC (%)	pH	Lactic-acid bacteria (colony forming units; Cfu g^−1^)	Yeasts (Cfu g^−1^)	Molds (Cfu g^−1^)	Total-bacteria (Cfu g^−1^)
Fresh maize	initial	625	27	5.8	1.9(0.2) × 10^8^	1.2(0.1) × 10^5^	ND	1.9(0.3) × 10^8^
	end			4.6	4.5(0.9) × 10^8^	5.6(1.8) × 10^5^	ND	2.1(1.3) × 10^8^
Fresh ryegrass	initial	425	45	6.1	3.0(1.7) × 10^5^	5.1(3.9) × 10^5^	2.2(0.7) × 10^6^	2.6(0.4) × 10^8^
	end			5.2	3.8(0.4) × 10^8^	1.6(0.5) × 10^5^	3.9(4.4) × 10^5^	1.4(0.2) × 10^8^
Ensiled maize	initial	625	35	4.0	2.5(1.3) × 10^9^	2.3(3.7) × 10^6^	ND	1.6(0.3) × 10^9^
	end			3.9	2.9(2.6) × 10^7^	6.8(7.5) × 10^4^	>1.0 × 10^3^	2.6(3.7) × 10^8^
Ensiled ryegrass	initial	425	43	5.2	3.0(0.2) × 10^6^	ND	ND	3.9(0.8) × 10^6^
	end			5.1	2.7(1.9) × 10^6^	ND	ND	6.0(2.8) × 10^6^

Most of the CO_2_ was produced during the anaerobic period, rather than the aerobic period. During the anaerobic phase, O_2_ remained depleted, but CO_2_ increased 3- to 5-fold, accompanied by a pronounced decline of pH (maize to pH 4.6 and ryegrass to pH 5.2). The decline in pH lagged the depletion of O_2_ in both cases (Fig. 2a,b), reflecting increasing anaerobic lactic acid production by lactic acid bacteria (LAB)^22^.

**Figure 3:**
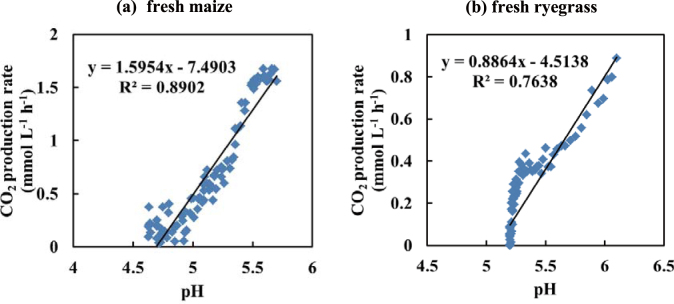
Correlation between pH and the rates of CO_2_ production during the anaerobic phase. Points represent hourly averages for (**a**) fresh maize (R^2^ = 0.8902, *p* = 6.04 × 10^−30^) and (**b**) fresh ryegrass (R^2^ = 0.7638, *p* = 5.60 × 10^−40^).

The rate of CO_2_ production was linearly related to pH for both fresh maize (Fig. 3a, R^2^ = 0.8902,  *p*= 6.04 × 10^−30^), and fresh ryegrass (Fig. 3b, R^2^ = 0.7638,  *p*= 6.04 × 10^−40^). When pH declined to 4.6 in maize and 5.2 in ryegrass, CO_2_ production and further changes in pH and *ΔP* were greatly reduced (Figs 2 and 3). This reflects suppression of microbial activity and further acid production by the reduced pH, despite the modest increase in counts of LAB observed in maize (Table 1), and the substantial increase observed in ryegrass.

*ΔP* exhibited a modest initial decline to a negative peak of −2.6 kPa in maize and −1.8 kPa in ryegrass, followed by a large increase to +45.4 kPa in maize and +19.1 kPa in ryegrass (Fig. 2a,b). Reasoning from the ideal gas law, observations of *ΔP* < 0 could result either from a loss of mass in the gas phase or from a decline in T_si_, or a combination of both. Similarly, *ΔP* > 0 could result from an increase of gas phase mass and/or elevation of T_si_. The increase in *ΔP* beginning as O_2_ declined to near zero (Fig. 2a,b) closely tracked the time course of rising CO_2_ in each test silo, demonstrating the essential role of gas phase CO_2_ production exceeding O_2_ consumption (Fig. 2a,b; upper panels). The CO_2_ produced in the fresh maize system was considerably greater than that in the fresh ryegrass, reflected in both CO_2_ and *ΔP* (Fig. 2a,b).

The relationship between CO_2_ and *ΔP* was biphasic in both systems (Fig. 2c,d). Piecewise regression analysis revealed strong linear relationships in both phases for maize (Fig. 2c; R^2^ = 0.9220, *p* = 5.94 × 10^−40^ for y_1_ and R^2^ = 0.9952, *p* = 7.12 × 10^−80^ for y_2_) and for ryegrass (Fig. 2d; R^2^ = 0.8856, *p* = 3.57 × 10^−61^ for y_1_ and R^2^ = 0.9952, *p* = 3.70 × 10^−90^ for y_2_).

An increase in T_si_ was observed in both systems during the aerobic phase (Fig. 2a,b). This stabilized at an elevated level in maize, but declined to original values in ryegrass during the anaerobic phase. More heat was generated during aerobic than during anaerobic respiration^8,23^. At the end of the ensiling process (about 100 h), pH was low, *ΔP* was elevated, and all parameters were stable. The experimental system remained anoxic.

### Ensiled plant materials (Test-B)

**Figure 4:**
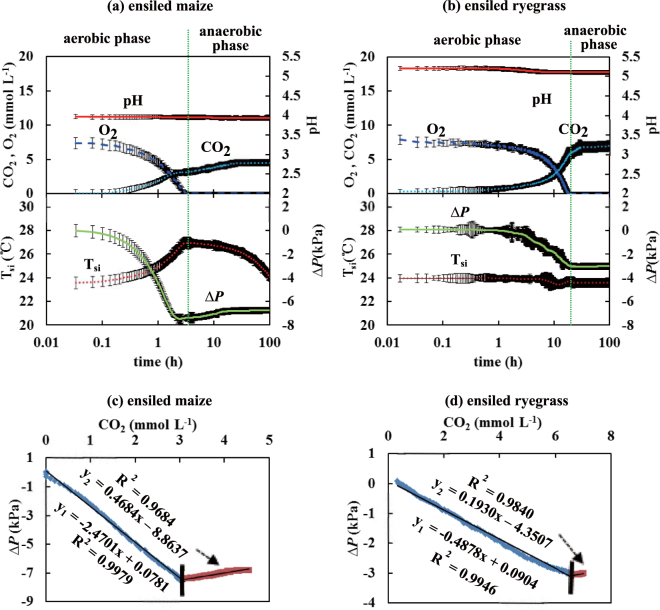
The time courses (means ± s.e.) of O_2_, CO_2_, pH, *ΔP* and T_si_ observed in the re-ensiled maize (Fig. 4a) and the re-ensiled ryegrass (Fig. 4b) during Test-B. The relationships between *ΔP* and CO_2_ shown as piecewise linear regressions during re-ensilage of fresh maize (Fig. 4c) (R^2^ = 0.9979, *p* = 3.30 × 10^−94^; R^2^ = 0.9684, *p* = 4.21 × 10^−87^) and re-ensiled ryegrass (Fig. 4d) (R^2^ = 0.9946, *p* = 6.90 × 10^−86^; R^2^ = 0.9840, *p* = 8.9 × 10^−89^) in Test-B.

The time courses of O_2_, CO_2_, pH, *ΔP* and T_si_ and relationships between *ΔP* and CO_2_ in Test-B were obtained with three repetitions (Fig. 4). The aerobic period for ryegrass was longer than for maize. This may be partially attributed to the lower packing density and resulting greater porosity in the ensiled ryegrass, leading to more O_2_ molecules entrapped but fewer microorganisms within the test silo^9^. The initial populations of LAB in ensiled ryegrass (10^6^ Cfu g^−1^) and total-bacteria (10^6^ Cfu g^−1^) were significantly lower than in ensiled maize (10^9^ Cfu g^−1^ and 10^9^ Cfu g^−1^, respectively; Table 1). Molds were initially below detection threshold (10^2^ Cfu g^−1^) in maize, while both yeasts and molds were below detection threshold (10^2^ Cfu g^−1^) in ryegrass throughout the experiment (Table 1). The fresh maize and the ensiled maize had similar (10^8^ Cfu g^−1^) microbial populations, whereas the fresh ryegrass had higher (10^8^ Cfu g^−1^) populations than the ensiled ryegrass (10^6^ Cfu g^−1^; Table 1).

In contrast to the biphasic time courses observed in Test-A (cf. Fig. 2), the CO_2_ concentration in both ensiled materials was lower than in the same material in the fresh state (cf. Figs 2 and 4) and *ΔP* only exhibited negative values throughout Test-B. The CO_2_ concentration in the ensiled ryegrass was greater than in the ensiled maize (Fig. 4), even though the microbial counts in the ensiled ryegrass were lower (Table 1). As a result, the decline to negative *ΔP* in the ensiled maize was greater than the ensiled ryegrass.

**Figure 5:**
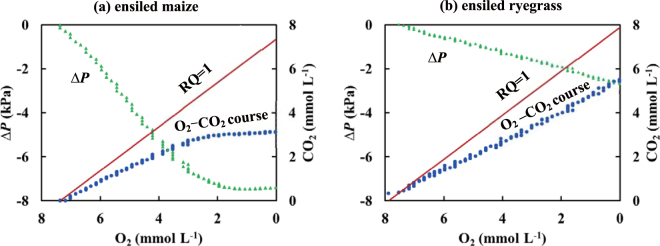
The apparent respiratory quotient (RQ) determined from the simultaneous measurements of O_2_ and CO_2_ for the re-ensiled maize (Fig. 5a) and for the re-ensiled ryegrass (Fig. 5b) during Test-B (aerobic phase). The reference line of RQ = 1 is the expected value from carbohydrate metabolism. The O_2_-CO_2_ course below RQ = 1 (i.e. actual RQ < 1) in Fig. 5a or Fig. 5b reflects the CO_2_ not detected by the sensor and apparently dissolved in the aqueous phase. Note the declining axes for O_2_.

As with the fresh material, observations of *ΔP* < 0 reflected a decline in gas phase mass inside the test silo. The simultaneous measurements of O_2_ and CO_2_ over time indicate that the respiratory quotient (RQ, ratio of mole CO_2_ evolution per mole O_2_ uptake^24^), was significantly below 1.0 for both ensiled materials (Fig. 5a,b; blue line). While aerobic carbohydrate metabolism exhibits a theoretical RQ = 1.0, i.e. 1 mole of CO_2_ evolved per 1 mole of O_2_ consumed^5,25^, in Test-B the observed RQ < 1.0 suggested the concurrence of CO_2_ production by aerobic respiration and simultaneous partial CO_2_ dissolution in interstitial silage water. This process was dominated by CO_2_ dynamics since the solubility of CO_2_ in H_2_O (39.2 mmol L^−1^ at 20 °C) is much greater than that of O_2_ (1.38 mmol L^−1^)^26^.

The relatively low CO_2_ concentrations in both ensiled substrates are likely to reflect several interacting factors. First, the produced CO_2_ was partially dissolved in the interstitial silage water. Second, after the fermentation (anaerobic phase), the residual plant sugar in the ensiled materials was sufficiently low that the CO_2_ produced from microbial respiration was minimal. In addition, microbial metabolic activity was further depressed by low pH^5,8,18,21^. The greater CO_2_ in ensiled ryegrass than ensiled maize reflects the lower density and mass of silage, greater porosity and volume of O_2_ entrapped, despite the similar O_2_ concentrations, and less available silage water for gas dissolution. Therefore, more CO_2_ was produced through aerobic respiration and less CO_2_ was dissolved in the interstitial silage water.

As observed for fresh material (cf. Fig. 2c,d), strong biphasic linear relationships were observed by piecewise regression between *ΔP* and CO_2_ in both ensiled maize (Fig. 4c: R^2^ = 0.9979, *p* = 3.30 × 10^−94^ for y_1_ and R^2^ = 0.9684, *p* = 4.21 × 10^−87^ for y_2_) and ensiled ryegrass (Fig. 4d: R^2^ = 0.9946, *p* = 6.90 × 10^−86^ for y_1_ and R^2^ = 0.9840, *p* = 8.9 × 10^−89^ for y_2_). In both fresh and ensiled materials, the goodness of fit demonstrated that *ΔP* varied primarily with CO_2_, rather than with T_si_.

## Discussion

In Test-A, *ΔP* < 0 was observed over a quite short period (around 5 h) following introduction of fresh plant material. The transition of *ΔP* from negative to positive indicated that the CO_2_ produced by microbial respiration rapidly exceeded the capacity for CO_2_ dissolution, while high pH allowed sustained metabolic activity during the early stages of ensilage. Therefore, *ΔP* is a potentially useful indicator not only of CO_2_ dynamics, but also of microbial activity. On the other hand, the solubility of CO_2_ in H_2_O is a function of numerous interacting factors, including pH, partial pressure of CO_2_ (Henry’s law), ambient temperature (van’t Hoff equation), interaction between internal gas pressure and temperature (the ideal gas law), sugar content in water and water content in the substrate^26–30^^,^. Adding further complexity, dissolution of CO_2_ in water is exothermic^27^, contributing to the observed increases in both T_si_ and *ΔP*. Since dissolution of CO_2_ in water is the outcome of the chemical reaction, CO_2_ + H_2_O ↔ H_2_CO_3_^26^, the decline of silage pH over the silage-making process resulted not only from the anaerobic fermentation of plant sugars such as glucose and fructose to lactic acid by LAB, but also from the dissolution of CO_2_ in water. As a result, the time courses of net CO_2_ production and *ΔP* are not readily simulated using current methods. Here we characterize the trajectory of variation in *ΔP* (*ΔP* = 0) → (*ΔP* < 0) → (*ΔP* > 0) → (*ΔP* < 0) throughout the silage production process. Observations of *Δ* *P* < 0 indicate apparent RQ < 1, reflecting the sequestration of CO_2_, and *ΔP* > 0 reflects the greater amount of CO_2_ produced during anaerobic respiration. While other gases may contribute to these effects, CO_2_ dominates because concentrations of H_2_, CH_4_ and N_2_O in silage are very low compared to N_2_, O_2_, and CO_2_^5,18,19,31–33^, and H_2_ and N_2_ exhibit low solubility in silage water.

For a field-scale bunker silo during feed-out phase, the working face is exposed to air as the silage is unloaded for feeding to livestock. This results in re-ensiling of the remaining material and unavoidable aerobic deterioration in the surface zone as O_2_ penetrates into the silage. With the two ensiled plant materials, Test-B provided an analog of these biochemical and physical processes of aerobic respiration and partial dissolution of CO_2_ in the interstitial silage water. Here we infer changes in the dissolved CO_2_ in the silage water, but carefully evaluate the role of gas phase advective transfer associated with CO_2_ dissolution. Our unique sensor combination allowed us to follow O_2_ and CO_2_, *in situ*, while tracking the variations of *ΔP* and T_si_ simultaneously, with *ΔP* employed as an indicator of the net CO_2_ dynamics based on the ideal gas law. For this reason, Test-B was conducted under gas-tight and constant volume conditions. Most real-world silage-making processes, including the maize bunker silo and ryegrass bale system used here, rarely exhibit this level of seal^5,17,34^. Our experimental design revealed the substantial and contrasting levels of *ΔP* generated by two common plant materials at the early ensiling stage and in the ensiled state. The rigorous vacuum sealing of our test silos allowed *ΔP* to vary over a range of −7.5 to + 45.4 kPa for maize and of −3.1 to 19.1 kPa for ryegrass.

To our knowledge, we provide the first evidence of the physical and chemical interactions involved in the production of CO_2_, its partial dissolution in silage water, and resulting negative and positive pressures, depending on stage of ensilage. The *ΔP* > 0 (‘balloon’) phenomenon has been reported previously^17–19,35^. One study^19^ observed an increase in O_2_ and N_2_ but a decrease in CO_2_, measured at the end of the eight-day fermentation phase, and inferred a reversal of gas movement from efflux to influx, as a result of the reversal of *ΔP* from positive to negative. However, fundamental processes and causative factors were not explored. Our multi-sensor measurements provide insight into these parameters with essential interactions and dynamics, and the resulting relationships between net CO_2_ production, O_2_ consumption, and variations in *ΔP*, pH and T_si_.

## Conclusions

*In situ* tracking of O_2_ depletion, CO_2_ production and dissolution, pH lowering-processes and *ΔP* dynamics has been realized during early and late phases of silage production, using multi-sensor techniques. CO_2_ production was much greater from anaerobic than aerobic processes. The *ΔP* exhibited both positive and negative values, indicative of net values of CO_2_ production and dissolution. For the two common silage materials tested here, the resulting *ΔP* is of the order of tens of kPa. Since the negative *ΔP* allowed more O_2_ entry through the silo face, risk of aerobic deterioration in the feed-out phase could be potentially higher than anticipated. These interactions are diagnostic of the previously unknown behavior during ensilage and re-ensiling processes. These results suggest that both gas diffusive and advective transfer play equally important roles in governing the complex temporal progression of inward and outward gas fluxes to and from the silage interior. Future efforts to develop simulation of the ensilage process should incorporate both Fick’s law and Darcy’s law. The fusion of our data with improved process models will support development of improved silage management.

## Methods

### General information on the sensors used

Table 2 lists general information of all sensors used for this study. These sensors employ different principles, each providing sufficient temporal response (<1 min.) to meet the data sampling requirement. This level of performance is critical for tracking O_2_ as it is converted to CO_2_, in order to interpret the resulting *ΔP* and T_si_ dynamics. Cross calibration of the O_2_ and the CO_2_ sensors were carried out in advance of the experiment^36^.

**Table 2:** Sensor specifications.

Parameter	Sensor-Type	Manufacturer	Range/Accuracy	Response (s)	Principle
Oxygen	KE-50	FIGARO Engineering Inc. Osaka, Japan	0–100% (vol.)/±2% of full scale (fs)	60	Electrochemistry
Carbon Dioxide	Prime 3	Clairair Ltd.Essex, UK	0–100% (vol.)/±3% fs	60	NDIR
Pressure	FDA612SR	Alborn Mess-und Regelungstechnik Gmbh Germany	±1000 (mb)/±0.5% of final value in range	1	Piezo-resistive pressure sensor
Temperature	FTA15 NiCr-Ni ZA9020FS	Alborn Mess-und Regelungstechnik Gmbh Germany	−40–160 (°C)/±0.1 °C	2	Thermocouple
pH	BlueLine 21 Ph	Alborn Mess-und Regelungstechnik Gmbh Germany	2–13/±0.3	<60	Potentiometric analysis

During each test period the pH sensor tip (diameter 2 mm) was enclosed in a small piece of well-wetted porous plastic to improve hygroscopic contact with the test material. The porous plastic was rinsed with deionized water and then dried naturally. Initial pH was determined using a standard protocol for silage^37^, by extracting 25 g of maize or ryegrass material with 0.225 L deionized water in a glass vessel for 30 min. This provided an initial reference value for comparison with the initial value of the inserted pH sensor when encased in the porous plastic wetted with the same solution. In addition, the pH sensor was repeatedly calibrated against three buffer pH-solutions (ZB98PHPL4, pH = 4.0, ZB98PHPL7, pH = 7.0, and ZB98PHPL10, pH = 10.0; AHLBORN GmbH, Germany) at 20 °C prior to and after the experiment.

### Experimental test silo

Two PVC cylinders (inner diameter: 370 mm, height: 600 mm, wall thickness: 15 mm) together with a lid (diameter 400 mm, thickness 20 mm) were used as the experimental silos (Fig. 1). To make the cylinder gas-tight, a rubber membrane (diameter: 400 mm, thickness: 10 mm) was affixed by a layer of vacuum grease to the inside of the lid, which was secured using six equidistantly-spaced fasteners. Five access ports through the lid and membrane allowed connection of the sensors inside the cylinder to a data logger (ALMEMO-2890-9, 9-channel, AHLBORN GmbH, Germany) placed outside. After each cable was placed through a hole, the space around the cable and all cable terminals were sealed with vacuum grease. The data logger operated at a sampling interval of 3 min throughout the experiment.

### Microbial analyses

Thirty grams of silage were suspended in 270 ml of ¼-strength Ringer solution (2.25 g l^−1^ NaCl, 0.105 g l^−1^ KCl, 0.06 g l^−1^ CaCl_2_, 0.05 g l^−1^ NaHCO_3_) (Merck, Darmstadt, Germany) and homogenized in a mixer for one minute. From this suspension, total bacterial counts were analyzed on plate-count agar (5.0 g l^−1^ enzymatic digest of casein, 2.5 g l^−1^ yeast extract, 1.0 g l^−1^ glucose, 15 g l^−1^ agar, pH 7.0) (Merck, Darmstadt) after aerobic incubation at 30 °C for 2 days. Lactic acid bacteria (LAB) were quantified from this suspension on de Man, Rogosa and Sharpe (MRS)-agar (10 g l^−1^ enzymatic digest of casein, 10 g l^−1^ meat extract, 4 g l^−1^ yeast extract, 20 g l^−1^ glucose, 2 g l^−1^ K_2_HPO_4_, 1.08 g l^−1^ tween 80, 2 g l^−1^ triammonium citrate, 5 g l^−1^ sodium acetate, 0.2 g l^−1^ MgSO_4_ × 7 H_2_O, 0.05 g l^−1^ MnSO_4_ × 4 H_2_O, 14 g l^−1^ agar, pH 5.7) (Merck, Darmstadt). MRS agar plates were incubated anaerobically using an Anaerocult system (Merck, Darmstadt). Yeasts and molds were detected using yeast extract glucose chloramphenicol (YGC)-agar (5.0 g l^−1^ yeast extract, 20.0 g l^−1^ glucose, 0.1 g l^−1^ chloramphenicol, 14.9 g l^−1^ agar, pH 6.6) (Merck, Darmstadt). These agar plates were incubated at 25 °C for 3 days.

### Test-A

This was an analogue to the ensiling phase using fresh chopped whole shoots of maize (*Zea mays*) including the cobs (dry matter content, DMC of 27%) and fresh-cut ryegrass (*Lolium perenne*; DMC of 45%). The resulting biomass was finely chopped and packed to high bulk density (BD) in separate cylinders (maize: 625 kg m^−3^, ryegrass, 425 kg m^−3^) using a hydraulic ram (360 mm diameter circular foot), in approximately six layer increments to ensure uniform density. Five holes (diameter 10 mm, depth 100 mm) through the top layer of silage, aligned with the holes in the lid, were drilled using a hole-saw for the installation of O_2_, CO_2_, *ΔP*, pH and T_si_ sensors.

### Test-B

Test-B used previously ensiled maize with DMC of 35% and ryegrass with DMC of 43%. Ensiled maize (harvested and ensiled autumn 2015) was obtained from 20 cm behind the freshly cut silage face in a concrete bunker silo (40 × 6 × 3.5 m), located at the Frankenforst research farm (University of Bonn, Germany). Ensiled ryegrass was obtained from a silage bale (harvested and ensiled autumn 2015), which was stored outside, wrapped in four layers of plastic film (thickness: 0.025 mm). The maize and ryegrass silages were re-ensiled in the test silos, and packed with the hydraulic ram to a high BD of 625 kg m^−3^ and 425 kg m^−3^, respectively. The sensors were installed as for the fresh materials. To achieve similar temperatures for the two experiments and to minimize ambient temperature fluctuations, both tests (each with three repetitions) were conducted in a large insulated workshop in the summer of 2016.
